# Making Psychology “Count”: On the Mathematization of Psychology

**DOI:** 10.5964/ejop.4065

**Published:** 2023-02-28

**Authors:** Simon Nuttgens

**Affiliations:** 1 Faculty of Behavioural Sciences, Yorkville University, Fredericton, NB, Canada; Dublin City University, Dublin, Ireland

**Keywords:** qualitative research, quantitative research, history of psychology, research methods, epistemology

## Abstract

Beginning in the late 18th century and continuing through to the mid-20th century, a movement was undertaken by psychology’s pioneers to establish a mathematical basis for research modeled after the physical sciences. It is argued that this movement arose through sociopolitical pressures to legitimize psychology as an independent discipline; demarcate its disciplinary boundaries within academia; and distinguish psychology from philosophy and spiritualism. It is argued that an ahistorical view of how the quantitative paradigm gained ascendancy leaves it largely unquestioned and unchallenged within mainstream psychology. Because of this, qualitative research has endured a long and continuing struggle to gain disciplinary recognition and epistemological parity. It is proposed that despite being sidelined by decades of quantitative hegemony, qualitative research has a long history in psychology and in the last 40 years has continued to prove itself as a necessary and valuable contributor to research in psychology.

“Behind psychological research lies an ideological support structure. By this I mean a discipline-wide, shared system of beliefs which, while it may not be universal, maintains both the dominant methodological practices and the content of the dominant methodological educational programs.” (Michell, 1997)

Back in the 1850s at a time when psychology was still in its infancy, Gustav Fechner (1801–1887) lay in bed puzzling over how he might scientifically establish a relationship between mind and body. The answer that came to him involved two components. First, he would need to assign values to differential magnitudes of mental sensations. Once achieved, he would then relate values to a systematically varied physical stimulus. This psychophysical solution to the mind-body problem is recognized as the first psychological theory capable of being experimentally tested that took mathematical form (Hearst, 1979). Fechner’s theory, which came to be known as the *Fechner-Weber function,* addresses the relationship between the physical magnitudes of a stimulus and the perceived intensity of a stimulus. The legacy of Fechner's pursuit to quantitatively answer the mind-body question set in motion what became the dominant and near-exclusive research paradigm within psychology: quantitative experimental research. In this paper I contend that the hegemonic ascendency of quantitative research within psychology was largely conceived through the zeitgeist of the late 19th –to mid–20th century at which time the fledgling discipline sought to legitimize itself as a science (Brinkmann et al., 2014; Eisner, 2003). Although such legitimatization was realized, it came at the expense of qualitive research, which despite its suitability to redress some quantitative shortcomings, has been significantly overshadowed by its methodological counterpart. The growth of qualitative research in recent years suggests that a broader, more inclusive approach to psychological research is gaining momentum.

## Calculated Beginnings of Quantitative Psychology

The great dream of the “new psychology” was fueled by the promise of the scientific method to illuminate all fixtures of human life (LeShan, 1990). Indeed, evidence of scientific progress during the nineteenth century confronted both layperson and academic at virtually every turn:

“New wonders were appearing every day and what was considered sorcery one day was commonplace and understood by school children thirty years later. The culture looked to the men in white coats, to the laboratories, to solve all of the ills of the human condition and to save us from poverty, hunger, backbreaking toil, cold and darkness.” (LeShan, 1990, p. 13).

Given the many visible triumphs of the physical sciences, it is not surprising that members of the nascent discipline of psychology would embrace as its method one closely modeled after the physical sciences. To do so would be both prudent and timely considering less credible alternatives available at the time (Bakan, 1996; LeShan, 1990). It was in the universities that science established its epistemological stronghold. Since the time of Newton, universities turned sharply in support of the natural sciences whose contributions to human betterment were highly valued. Accordingly, within the university setting these disciplines enjoyed privileged status and ample resources to conduct their activities. In contrast, within the late nineteenth century university, psychology was a junior member, most always placed as a subordinate within the department of philosophy (Bakan, 1996; LeShan, 1990). To elevate its status, psychology required a method that would simultaneously elevate its standing and legitimize its worth. Use of a scientific method modeled after the physical sciences would serve this purpose.

The impetus to secure psychology within the domain of a legitimate science was also related to a thriving interest in spiritualism present in the mid–to–late 1800s. Coon (1992) proposed that psychologists at that time feared that the investigation of supernatural phenomena might jeopardize the already tenuous designation of psychology as a science. Interest in the in the occult, telepathy, and related phenomena at the turn of the century was viewed by many psychologists as a threat to their discipline’s status as a science on par with the physical sciences. However, it was not easy to displace the interest in spiritualism, as both the public and some eminent psychologists, notably William James, considered the investigation of supernatural phenomena to be equally important to the study of more objective areas of research. Eventually, investigations into the paranormal were taken up by experimental psychologists if for no other reason to prove their fraudulence or offer naturalistic explanations (Coon, 1992). The turn toward scientific explanation can also be understood within the tide of secularization present at the turn of the century. With the encroachment of secularism on the religious worldview, science was increasingly used to explain what in the past would have been understood through church doctrine and scripture (Coon, 1992; Danziger, 2000).

## The German Origins of Quantitative Psychology

Eighteenth century Germany may be considered, perhaps ironically, as the birthplace of quantitative psychology. Ironically because it was Immanuel Kant's admonition that psychology could never truly be a scientific discipline that led his adversaries to want to prove him wrong, thus initiating a pull toward mathematization (Leary, 1978).

Kant believed that to be scientific a field of inquiry had to be amenable to mathematical investigation. In Kant's view, science required both rational and empirical components, with experience providing the empirical component and mathematics, when applied to the empirical data, providing the rational. However, Kant believed that mathematics could never be applied to psychological phenomena, and thus argued that psychology must necessarily remain non-experimental. In light of his position, Kant suggested that psychology make use of a different methodology, one that was anthropologic in nature. Such a methodology “could become more useful to mankind [sic] if it would forsake its traditional introspective method and begin to make systematic observations of men and ‘women in the world’ as they behave and interrelate with their fellow citizens” (Leary, 1978, p. 115). Leary’s Kantian-informed alternative is, of course, notably consistent with the qualitative paradigm; however, the resolve to make psychology an experimental science quashed Kant’s admonition until qualitative research began to build stature in the latter part of the twentieth century.

Johann Friedrich Herbart (1776–1841) moved first to prove Kant wrong by proposing that psychological phenomena of differing intensities over time could be assigned numerical value, and then, based upon an equilibrium model, be described in terms of precise mathematical equations. The problem with Herbart's method was that he assigned arbitrary numerical values to his intensity data and, hence, such values were not measured according to any objective standard (Leary, 1978).

Where Herbart left off, Gustav Theodore Fechner continued. Fechner retained Herbart's empirical methodology, though abandoned his questionable mathematics for a quantitative approach that he viewed as mathematically rigorous. Fechner, a physicist, directed his early experimentation toward verifying the mathematical theory of galvanic electricity. This work by Fechner necessarily involved the application of precise measurement and experimental control consistent with the requirements of the natural sciences. It was through such undertakings that Fechner thoroughly familiarized himself with a powerful quantitative “literacy” which he then applied to his research on the mind-body problem: “He brought to psychology in its prenatal state a contagious devotion to quantitative experimental rigor and to the hypothetico-deductive approach” (Marshall, 1990, p. 47).

Fechner believed that true mathematical descriptions are always approximations, yet he also believed that these approximations could become increasingly accurate and sophisticated. Thus, Fechner's goal was to render mathematics an accessible tool for scientists to use in all their inquiries, regardless of whether the subject matter was organic or inorganic (Marshall, 1990). In his paper, *Outline of a New Principal of Mathematical Psychology*, Fechner (1851/1987) dismissed the Herbartian method of measuring mental phenomena, which, as noted earlier, relied on the application of arbitrary values to mental events, arguing instead for a mathematical psychology that could rely on the observation of physical phenomena. Here Fechner (1851/1987) devised a logarithm based on various observations that show:

“How it comes about that mental phenomena, though on the whole always proceeding in parallel to physical phenomena and displaying corresponding modifications and turning points, are not proportional to the absolute magnitude of physical activities.” (p. 207)

In his paper, *My Own Viewpoint on Mental Measurement*, Fechner (1887/1987) argued for a general principle of mental measurement in which *n* magnitudes judged to be equal may be added and judged equal to the sum of a magnitude *n* times as large as the individual magnitudes. This, Fechner claimed, was the principle for all physical measurement and as such should be the basis for all mental measurement. It is evident that Fechner recognized the limitations of his own perspective when he writes “Whether or not it has wider application need not concern us here, as long as we are merely interested in the possibility and justification of mental measurement *as such*” (original italics; p. 213). Fechner further acknowledged that in keeping with the field of physics, the use of his general principle of mental measurement was problematic because the equality of two or more magnitudes could never be known with absolute precision. Fechner resolved this difficulty through his use of averages established through repeated measures.

At the time, Fechner's attempt to transform human mental activity into measurable form gathered considerable praise, notably from Wilhelm Wundt. Whereas Fechner was primarily interested in elucidating the laws of association, Wundt's desire was to examine more completely the aspects of consciousness including feelings, images, dreams memories, attention, and movement (Robinson, 1976). According to Wundt, this could only be achieved through the experimental study of the contents of consciousness as reported observations of internal events. Although he is routinely referred to as the father of experimental psychology (Boring, 1950), Wundt held a narrow view of what could be meaningfully subjected to experimental investigation; hence, even though he strongly promoted experimental psychology, he also declared that much of human experience lay outside of its purview (Blumenthal, 2002). Accordingly, Wundt’s research approach also included a decidedly qualitative approach, which he referred to as *Völkerpsychologie*. For Wundt, human experience was inextricably entwined with language and history and thus could only be fully understood through cultural products such as customs, myth, and religion (Brinkmann et al., 2014). Given the amount of writing that Wundt’s committed to his Völkerpsychologie (10 volumes), he clearly viewed this arm of his research as an integral component of psychological investigation (Brinkmann et al., 2014; Wertz, 2014).

Fechner's theories had a significant influence on experimental psychology in general, and quantitative psychology in particular (Hearst, 1979). Though it is difficult to identify any single intellectual disciple of Fechner's (i.e., someone who was directly taught by him and later gained prominence), through rising to Kant's challenge to mathematize psychology, Fechner enabled the fledgling discipline to distance itself from its philosophical leanings, and instead pivot its methodological paradigm toward the revered natural sciences.

## Galton in England: A New “Breed” of Quantification

Sir Francis Galton (1822–1911) is consistently noted as a central figure in the founding of modern psychology. At age twenty-two Galton inherited a substantial sum of money from his father who had gained wealth in the banking industry. This afforded Galton the luxury to devote his entire life to personal interests, of which he had many. Despite not possessing an aptitude toward mathematics, one of Galton's most enduring traits was a fascination with measurement (Cowan, 1972). Most of Galton's published papers had some concern with counting and measurement, whether this was determining the accuracy of geographical instruments or precisely recording weather conditions. However, Galton’s general interest in measurement shifted upon reading *On the Origin of Species*, published by his cousin, Charles Darwin. Galton was fascinated by this work, taking from it the notion that hereditary could form a basis for improving humankind, believing that “over a period of time men could be bred for intelligence and character in precisely the same way that animals are bred for strength or agility” (Cowan, 1972). Galton's interests in measurement thus turned toward individual differences in psychological attributes and its companion territory of eugenics.

To better understand the direction and tenor of Galton's psychological pursuits, it is necessary to consider the political and economic forces present in 19th century England (van Strien, 2005). As will be shown, such forces led to a social climate favourable to the quantitative study of individual differences and inherited ability. Galton lived and conducted his affairs in England at a time when the expansive undertakings of the colonial empire were at its peak, a time when even amid oppressive social concerns, optimism for the perfectibility of society thrived. Buss (1976) contends that the growing division of labour in 19th century England was a social phenomenon that invited use of a scientific explanation for individual differences. This, along with Galton's familiarization with Darwin's theories of natural selection and evolution, set the stage for an approach to understanding human difference that would “explain and justify the hierarchically structured occupational groups and attendant social inequalities” found in Victorian England (Buss, 1976, p. 52). This undertaking was necessary because the prevailing democratic liberalism, with its stress on individual freedom and development, was inconsistent with the existing social structure and division of labour that prevailed in England at the time. Reconciliation of this discrepancy was sought through recourse to “scientifically” derived findings of inheritable differences in individual ability. The view that individual differences could be shored up and enumerated through quantitative methodology, was entirely amenable to a historical epoch enamored with capitalist values where quantification already played a central role in determining and understanding economic vicissitudes: “Just as it is possible to measure and quantify man's products, it is possible to quantify man [sic] himself” (Buss, 1976, p. 53).

In his first book, *Hereditary Genius*, Galton attempted to demonstrate that greatness tended to run in families and that this ability would produce a normal curve within the population of all British men. To accomplish this, Galton drew upon Quetelet's notion of the error curve, though without utilizing its probabilistic qualities (Cowan, 1972). This next step was to formulate a mathematical law of hereditary based upon statistical units of deviation. Galton achieved this through the study of sweet-pea data. Galton noticed that there was an imperfect relationship between inter-generational transference of pea size, especially in extreme cases where the filial pea might be of significantly different proportion than its parent. One might presume that this finding would have been taken as evidence that hereditary was not the stable law Galton believed it to be. Not so. In response to this contrary finding Galton proposed that the tendency for exceptional cases to regress toward the population mean did not suggest that traits were not inherited, but rather that trait-based anomalies should be interpreted as statistical artifacts explainable by error law. Thus, Galton proposed that ability was inherited, that in general the ability of one's parents is associated with the ability of one's child, with the caveat that extreme examples would likely regress toward the mean according to mathematical laws (Cowan, 1972). The need to confirm his ideas beyond evidence afforded by his sweet-pea data lead Galton to set up “anthropomorphic laboratories” in 1884 at the International Health Exhibition and in 1904 at the University of London. These laboratories measured physical characteristics such as height, weight, span, breathing power, strength of grip. This data provided Galton with the raw material needed to establish his statistical discoveries (Cowan, 1972). In 1888 Galton extended his theory of regression through the realization that a regression coefficient could determine the relationship between any two variables regardless of whether they were concerned with the hereditary process or whether they were measured by the same unit. This became the basis for modern day correlation studies (Cowan, 1972).

According to Cowan (1972), regression and correlation were Galton’s two most important contributions, not only to psychology, but to the history of science. As such, both were firmly rooted in the social context of Victorian England, a period when science was used to legitimize social hierarchy. Galton’s contribution to statistical method is an apt example of how methodological innovation is subservient to social expedience. Another example that follows both in time and character is that of one of Galton's most ardent admirers, Charles Spearman. Lovie (1991) extends the thesis that Spearman's invention of factor analysis from its inception was not the neutral technique that its early proponents made it out to be, but rather:

“Had been developed to prove the existence of a previously postulated psycho-social reality. For Spearman and his numerous followers factor analysis was there to give the scientific and statistical cachet to the idea that intelligence was hierarchical in nature. It was not, therefore a device for uncovering any old structure, but a means of demonstrating a particular one.” (p. 243).

The wedding of mathematics and scientific method in the development of a quantitative methodology for psychology is inextricably woven into the social and political context in England. In North America a similar process was taking place, however, in this case it had to do with upstart universities rather than intelligence and eugenics.

## Mathematizing Psychology in America

In turn of the century North America the statistical movement in psychology was gaining momentum through the efforts of James McKeen Cattell (1860–1944). In 1886 Cattell became the first American to graduate with a doctoral degree in psychology from Wundt's Leipzig laboratory, and thus not surprisingly firmly believed that mental phenomena were amenable to scientific investigation. Following his stay at Leipzig, Cattell made brief stops at the University of Pennsylvania and Galton's laboratory in England before settling for good at Columbia University.

Cattell spent little time as a laboratory scientist. Instead, he served fifty years as editor and publisher of the prestigious journal, *Science*, a position of authority that undoubtedly gave increased visibility to psychology within the broader scientific community (Benjamin, 1997). Though he had studied primarily under Wundt, it was Galton who had the greatest influence on Cattell. Sokal (1982) suggested that it was Cattell's visit to Galton's laboratory that gave him his scientific goal to measure individual differences in psychological functioning. In Cattell’s words:

“Psychology cannot attain the certainty and exactness of the physical sciences, unless it rests on a foundation of experiment and measurement. A step in this direction could be made by applying a series of mental tests to a large number of individuals. The results would be of considerable scientific value in discovering the constancy of mental processes, their interdependence, and their variation under different circumstances.” (Cattell, 1890, as cited in Benjamin, 1997, p. 142).

Cattell’s visit with Galton supplied him with a goal for psychology but not the methodology. Unlike Galton, Cattell showed little interest in analyzing variation itself; rather, Cattell preferred to follow the tradition set forth by Fechner of interpreting error as deviations from a true value. Perhaps more important than adherence to any specific approach to measurement was the overall role Cattell played in establishing statistics in psychology within North America. Camic and Xie (1994) propose that Cattell's influence, strong as it was, served part of a larger movement within the social sciences (anthropology, psychology, economics, and sociology) to secure themselves a spot in the new and expanding universities of late nineteenth century America. The need for a discipline to find its own scientific niche within an institutional setting is referred by Camic and Xie as boundary work, which they define as: “Activities aimed at demarcating a given scientific field from both nonscientific fields and neighbouring scientific fields in order to separate it from its competitors and enhance its legitimacy” (p. 776). Prior to the late 19th century and early 20th century, statistics-based scientific methodologies were largely absent from American universities. Adherence to statistical methods by Cattell and his contemporaries at Columbia University allowed for simultaneous differentiation and conformity to the more general scientific attitude prevalent at the time, thus enabling the boundary work necessary to galvanize psychology’s disciplinary status within American universities (Camic & Xie, 1994). Statistics eventually became an identifying mark for Columbia University, a mark that gave it a distinct edge in the highly competitive world of turn-of-the-century universities. Cattell’s legacy to establish statistics as a fixture of American psychology was further advanced by his students Edward Thorndike and Robert Woodworth, both of whom advocated a quantitative-statistical psychology, and both of whom joined Columbia's faculty after graduating.

## The Inferential Turn

As early as 1904, psychologists in the United States were conducting research that employed relatively sophisticated statistical analysis. For example, in a 1904 study Robert Yerkes discussed the use of various statistical measures such as standard deviation, mean, median, and mode (Lovie, 1991). There is also evidence that decision rules were being used around this time to judge whether comparisons between mathematical means were significant, the general rule being that the ratio of the difference between two means should exceed some predetermined critical value. As is still the case with modern critical values based on probability distributions, there was at the time considerable debate over what ought to be considered an acceptable critical ratio value (Lovie, 1991). It was not until the 1940s, however, that inferential statistics firmly took hold within academic psychology in North America. Before this, inferential statistics were known about, though seldom used. It was Ronald Fisher who changed this.

Fisher first published ANOVA studies of agricultural topics in the 1920s. Fisher's book *Design of Experiments* was especially influential because, according to Gigerenzer (1989), it provided a “general methodological doctrine that could unify psychologist in all fields” (p. 207). This is what Fisher's statistical methodology achieved. Research psychologists seized upon Fisher's program of statistics and readily applied it to their own field as a means for testing and choosing among competing theories. This, conversely, was not what Fisher himself had intended: “Fisher, however, interpreted his procedure of null hypothesis testing, not as a means for practical decision making, but as a solution to the problem of induction and a rigorous method of scientific inference” (John, 1992, p. 145). In its modern usage, Fisherian statistics has become synonymous with the task of null hypothesis decision-making, again, contrary to Fisher's original intentions.

Fisher's method of statistical research was further misappropriated due to efforts of statistical psychologists who wrote textbooks during the 1950s. The form in which statistics in psychology takes today is derived from the shotgun marriage between Fisher's ideas and the competing ideas of Neyman and Pearson (Gigerenzer, 1989). This marriage was advanced by psychologists eager to incorporate techniques associated with perceived methodological rigor into their field to bolster its credibility within academia. As it happened, Fisher and his chief academic rivals, Neyman and Pearson, were in the late 1940s engaged in considerable debate over the practice of disproving null hypotheses as a method of inductive inference. This, of course, was the position advanced by Fisher. Alternatively, Neyman and Pearson argued that statistical power should be the governing rule when choosing between two hypotheses. Though both positions were offered by their respective proponents as distinct and competing statistical methods, textbook writers at the time chose to meld the two divergent approaches into a unified hybrid. To both parties—Fisher, and Neyman and Pearson—this would have been an irreconcilable union, yet join they did, forging a look for psychological statistics that continues today:

“It was not a time for alternatives: statisticians were eager to sell and psychologists were eager to buy *the* method of inductive inference. The statistical texts now taught hybrid statistics that neither Fisher nor, to be sure, Neyman and Pearson, would have approved.” (Gigerenzer, 1989, p. 208).

The hybrid statistics came to be viewed as a singular entity devoid of any recognition of the contributions of its original creators. The inconspicuous manner in which Fisher and Neyman’s and Pearson's statistical ideas were joined together is somewhat surprising considering a tradition set forth in which “controversies and alternative theories had always been the rule rather than the exception, and where the citation of authors, and more authors, had been common practice” (Gigerenzer, 1989, p. 209).

Ultimately, Fisher's most enduring contribution to psychology was the coupling of statistical methods with experimental research to an extent that today the two are treated as a single, inseparable, enterprise:

“Fisher has linked significance testing to experimental design, and the “inference revolution” was consequently a revolution in experimental design. This revolution has been so successful that it is often difficult for today's experimenters to imagine that “experiment” could mean something different from what Fisher taught.” (Gigerenzer, 1989, p. 208).

How did it happen that Fisher’s approach became so thoroughly enmeshed with experimental design? One answer has it that there was little alternative:

“Although Fisher's design could have been used without his statistics, his statistics did not mesh well with other designs. Thus, many alternative ideas of experiment did not survive the inference revolution, and Fisher's model won a monopoly.” (Gigerenzer, 1989, p. 209).

Tidying the loose bits of inferential statistics helped steer psychology away from its perceived subjectivity while also serving to cloak the theoretical discord that had been dogging psychology: “Broad theoretical commitments were dangerously divisive, and shared statistical methods did much to hold the field together”. (Porter & Porter, 1995, p. 211).

## Standard Errors

The quantitative imperative in psychology (Michell, 2003) has undoubtedly helped bring legitimacy, status, utility, and a form of methodological unity to the discipline (Danziger, 1985), and given how engrained a mathematically derived methodological base is within the discipline, it is difficult to imagine psychology without it. The problem, however, is that unlike British and European psychology, much of mainstream North American psychology continues to shield its methodological “self-consciousness” (Yurevich, 2009, p. 89) through a general (though certainly not exclusive) reluctance to recognize the merits of qualitative research. This cuts a sharp and decisive line through psychology’s epistemological core: on one side phenomena that are amenable to measurement, the other side phenomena that are not (Valsiner, 2000). Many epistemological concerns have been levied against the quantitative paradigm (e.g., those associated with positivist philosophy, laboratory experimentation, theory of measurement and error, objectivity, and reductionism), all of which culminate in the view that this paradigm leads to a “limited and distorted picture of phenomena involving human behavior” (McGrath & Johnson, 2003, p. 31). That qualitative research might enliven and enlarge an understanding of human behaviour is viewed by many (e.g., Harré, 2004; LeShan, 1990; Michell, 1999) as critical to psychology’s progress as a discipline. In what follows I highlight a few of the central criticisms that have arisen in response to the sweeping application of quantitative and statistical methods in psychology.

To begin, Michell (1997) has argued that the practice of measuring inner mental processes is inherently flawed, and that such an endeavour has mistakenly been construed as immune to the need for theoretical justification:

“It would seem that measurement has been mistakenly thought of by some philosophers as being an atheoretical, purely observational base upon which science's more theoretical structures stand. It is not. Measurement always presupposes theory: the claim that an attribute is quantitative is, itself, always a theory and that claim is generally embedded within a much wider quantitative theory involving the hypothesis that specific quantitative relationships between attributes obtain.” (Michell, 1997, pp. 358–359).

It is perhaps ironic that measurement in psychology is inconsistent with the original natural science definitions from which it is purportedly derived. Scientific measurement is defined as “the estimation or discovery of some magnitude of a quantitative attribute to a unit of the same attribute” (Michell, 1997). Key to this definition is the *additive* structure of the attribute that allows for meaningful ratios between magnitudes to be estimated or discovered. This can be contrasted with the definition of measurement in psychology offered by Stevens in the mid-1940s, which is still widely endorsed today: “Measurement is the assignment of numerals to objects or events according to rule” (Stevens, 1946, as cited in Michell, 1997, p. 360). The error of research psychologists who follow Stevens' definition is that they ignore that “the measurability of an attribute presumes the contingent (and therefore, in principle, falsifiable) hypothesis that the relevant attribute possesses an additive structure” (Michell, 1997, p. 361). By presuming that an attribute has a true additive structure in the absence of empirical evidence, one is drawn to the subsequent erroneous belief that “the invention of appropriate numerical assignment procedures alone produces scientific measurement” (Michell, 1997, p. 361).

That the above-stated discrepancy between the original definition of scientific measurement and psychology's own widely used definition remains virtually uncontested to this day, suggests that as a discipline we sometimes lack the degree of critical reflection necessary to progressively evolve as a discipline. Michell (1997) points to how this state may have arisen:

“From its inception, modern quantitative psychology was more concerned with the implementation of a quantitative program than with the pursuit of answers to fundamental scientific questions about its hypothesized quantities.” (p. 362).

Many branches of natural science are buttressed by the belief in empirical realism, which states that an independent natural world exists that is knowable through objective observational methods (Michell, 1997). In psychology we have come to view all human experience as amenable to empirical investigation.

“Modern empirical methods in the social and educational sciences are largely predicated on the *eye* as giving truth. The problem of modern science was to make observable that which was previously hidden. The survey instrument and the use of statistics, important inventions in the conduct of social sciences, reiterated the importance of the eye. Feelings, attitudes and perceptions were made public (observable) and comparable through the survey. Personal attributes became observable (or, in this case, countable) phenomena.” (Popkewitz, 1997, p. 20).

Here it is shown that the self must first be quantifiably objectified before the inner characteristic of the person can be treated as data (Popkewitz, 1997). A theoretical leap is taken in this instance whose assumptive status is rarely acknowledged or accounted for.

In addition to the difficulties that follow when attempting to fit human traits and experience into pseudo-quantitative categories, areas of concern also abound in the use of statistics. In his review of literature that addresses misunderstanding and misuse of statistics in psychology, John (1992) provides many examples of over-estimation and exaggeration of the “evidentiary value of statistic within psychology” (p. 144). John cites studies that show how academic psychologists mistake, misuse, or otherwise misinterpret the statistics used in their research. One telling example is a study by Cochran and Duffy (1974) in which 85% of 276 studies chosen for examination used inadequate sampling procedures. John also cites the well-known study by Peters and Ceci (1982) who resubmitted published articles to psychological journals that were subsequently rejected due to serious errors in statistical analysis. The identification of these concerns a few decades back were a harbinger of the current replication crisis in psychology (Wiggins & Chrisopherson, 2019).

As is the case with practices of measurement in psychology, statistical practices, though ready targets for critique, have largely gone unquestioned. John (1992) writes:

“In being naturalised within psychology, inferential statistics have become taken for granted as universal, coherent, noncontroversial collection of rule governed algorithms for the mechanisation of the production of conclusive knowledge, despite a history of continuous unresolved controversies over contradictory and irreconcilable philosophical and theoretical positions.” (p. 145).

Today, the use of inferential statistics has been likened to a rhetorical device that serves to establish epistemic authority within psychology (Danziger, 1985; John, 1992). This would be less problematic if it were not for the limitations that follow when a methodological bias restricts the scope of theories amenable to testing. In such instances the theory or elements thereof must either be transmuted to fit with the theoretical requirements associated with inferential statistics or be excluded from scholarly investigation. In this respect, methodological theory assumes the position of a “Hidden Hand that steers the research process as a whole in a certain direction” (Danziger, 1985, p. 3).

Central to the promotion of disciplinary competence in any field of practice is the role of education (Walsh-Bowers, 2002). John (1992) criticizes the teaching of statistics in its usual form because of it fails to acknowledge controversies and disputes, instead instructing students in the “cookbook application of various statistical techniques, so that students come unthinkingly to apply tests of statistical inference routinely as a kind of knowledge increase ritual” (p. 146). Proceeding in this manner can indoctrinate students into the quantitative imperative to a degree that even in the rare instances when they are introduced to qualitative methods, they remain skeptical of its import and place in psychology (Roberts & Castell, 2016). In psychology, statistics are almost exclusively associated with credible research (Danziger, 1985), to a degree that any research that does not use statistics is deemed unscientific and hence unacceptable to many journals:

“Psychological discourse strongly implies some inescapable connection between inferential statistics and scientific method and exploits the common belief in a status hierarchy of the sciences in which scientificity is equated with quantification and the use of mathematics.” (John, 1992, p. 147).

Research by Marchel and Owens (2007) is telling in this regard. When they examined the mission statements of fifty-seven American Psychological Association journals, only six were inclusive of qualitative research. It is unfortunate that presently the domination of the quantitative paradigm goes largely unquestioned and, hence, uncontested. One might legitimately wonder if the discipline’s insecurity at being scientific at the turn of the century has not followed psychology into the present. Outside of a few notable exceptions (e.g., educational psychology, cultural psychology psycholinguistics), the quantitative/statistical research paradigm continues to serve as psychology's dominant methodology, irrespective of limitations and criticism. Rennie et al. (2000) caution that defining knowledge by method recursively sustains the hegemony of the quantitative research paradigm within psychology departments. Efforts to sway this situation are held in check by institutional powers subservient to curricula oversight, hiring practices, and the requirements of granting agencies and editorial policies (Walsh-Bowers, 2002).

## Numbered Days

In much of psychology, quantitative research stands giant next to its qualitative counterpart (Jovanović, 2011). Dwarfed in stature, the prevailing assumption within many psychology departments is that qualitative research must, therefore, be inherently inferior in the domain of producing useful and credible knowledge (Danziger, 1985). A diminished view of qualitative research is aptly illustrated in the current empirically supported treatment (EST) initiative developed and promoted by Division 12 of the American Psychological Association (Society of Clinical Psychology). This initiative, which began in 1995, sought to strengthen the relationship between research and clinical practice through creating a list of ESTs that would indicate specific treatments for specific problems. Eighteen such treatments were identified, though not without considerable outcry from those who questioned the conceptual, political, and empirical platform that served to support the initiative. Regarding the latter, evidence for the purpose of this endeavour was based on a hierarchy of methods, with random clinical trials sitting sovereign on top, and case studies sitting proletarian on bottom. Accordingly, research of an idiographic nature was viewed as having marginal utility in the pursuit of clinical praxis.

Although the “which is best?” pursuit of the methodology wars has to a degree been supplanted by those who advocate a “different, yet both of value” position (Salvatore & Valsiner, 2010; Valsiner, 2000), there remains considerable room for qualitative research to catch up to quantitative research and flourish within traditional psychology departments (Rennie et al., 2000, 2002; Wertz, 2014).

It important to remember that the early days of psychology were much more methodologically diverse, with key figures such as Wundt and James arguing for a broad conceptualization of method and the likes of Freud and Piaget embossing their theories with observations and interview data. Such beginnings, however, appear to have been overlooked “by the official journals and handbooks of psychology (Brinkmann et al., 2014, p. 32). Even later into the twentieth century there were notable (and lasting) examples of qualitative research that stood apart from the emerging quantitative paradigm of the time. For example, in the 1930s John Dollard conducted field-based qualitative work on race and class relations by immersing himself as a participant-observer in the social life of small-town southern USA. Similarly, in the 1940s Muzafer Shirif and his research team engaged in observational research at a boys’ summer camp to learn about conflict and cooperation. Indeed, qualitative might have survived amidst the quantitative offensive if it were not for Word War II’s indelible influence upon German intellectual life. As discussed earlier, contributions by German intellectuals, such as Fechner and Kant, contributed greatly to the insurgent use of mathematical psychology. Yet, overall the German psychological scene coveted a diverse and inclusive epistemological, much more so than American psychology. Toomela (2007) writes of there being two psychologies in the pre-World War II era, a German-Austrian psychology characterized by insight (rather than prediction), holism, idiographic understanding, and qualitative description, and an American psychology characterized by experimental control, prediction, and atomism. While admittedly these differences are painted in broad strokes (individual exceptions could inevitably be found on both sides of the Atlantic), there were notable differences. Parity across these differences was disrupted by the Nazi rise and resulting German intellectual diaspora. Attempts to transplant the German methodological perspectives on foreign soils fell short, opening a wide door for the American approach to assume ascendancy (Toomela, 2007; Valsiner, 2006). Whereas pre-World War II American psychology proceeded without impediment, the German-Austrian tradition floundered. The value of the hard sciences, relative to the soft sciences, was further bolstered by the many stunning technological feats achieved during wartime (e.g., radar, the proximity fuse, computers, and atomic bombs (Solovey, 2004).

As alluded to earlier, qualitative research continues to slowly gain a foothold within academic psychology. While its presence dates to mid-18th century Germany, its current revival can be tracked to the 1960s (Brinkmann et al., 2014) at a time when humanistic and feminist movements began to puncture the fabric of social institutions, heralding an emancipatory counterculture that fostered “a more diversified, more differentiated, more open, flexible and inquisitive society” (Jovanović, 2011). Prior to the 1970s, the term “qualitative” was rarely used in academic research contexts (Karpatschof, as cited in Brinkmann et al., 2014). The number of qualitative research texts published by a major publisher (SAGE) beginning in the 1980s is indicative of its rising strength at this time. Between 1980 and 1987 only ten books were published by this publisher; this number rose dramatically to 133 between 1995 and 2002 (Gobo, 2005). Research by Rennie et al. (2002) noted a similar finding in their research examining the incidence of psychology database entries for terms related to qualitative research. Prior to the 1980s such entries were very rare, but then increased sharply in the 1990s. Recent initiatives also point to qualitative research’s strengthened position within academic psychology. For example, in Great Britain qualitative research has ostensibly attained a measure of equality with quantitative, as evidenced by the British Psychological Society requiring clinical psychology programs to offer training in both paradigms if they are to become accredited. The arrival of the journal “Qualitative Research in Psychology” in 2004 also did much to increase qualitative research’s visibility within mainstream psychology.

## Conclusion

This paper offers an overview of the contribution of pioneering psychologists who through conducting their affairs during a particular socio-historical context were able to establish within psychology a mathematical foundation for most of its research activities. The disciplinary dominance of the quantitative paradigm continues today despite qualitative research standing ready as a beneficial and complementary alternative. Psychology continues to align itself with a methodology long since removed from the important historical conditions that gave birth to it. Many within traditional psychology departments look upon qualitative research with either opposition or indifference (Camic et al., 2003; Stoppard, 2002). Few, according to Michell (1999) question the theoretical edifice upon which the quantitative paradigm has been built. Using measurability to define what “counts” as legitimate research needlessly excludes the investigation of certain types of psychological phenomena, namely, those that involve description, meaning, and story.
